# Low-pressure-responsive heat-storage ceramics for automobiles

**DOI:** 10.1038/s41598-019-49690-0

**Published:** 2019-09-18

**Authors:** Shin-ichi Ohkoshi, Hiroko Tokoro, Kosuke Nakagawa, Marie Yoshikiyo, Fangda Jia, Asuka Namai

**Affiliations:** 1 Department of Chemistry, School of Science, The University of Tokyo, 7-3-1 Hongo, Bunkyo-ku, Tokyo, 113-0033 Japan; 2 Division of Materials Science, Faculty of Pure and Applied Sciences, University of Tsukuba, 1-1-1 Tennodai, Tsukuba, Ibaraki, 305-8573 Japan

**Keywords:** Thermodynamics, Energy, Materials chemistry, Materials for energy and catalysis

## Abstract

The accumulated heat energy of a heat-storage material is typically released over time. If a heat-storage material could preserve its accumulated heat energy for a prolonged period, the applicability of such materials would be expanded greatly. Herein we report a newly fabricated heat-storage material that can store latent heat energy for a long period and release the heat energy upon demand by applying an extremely low pressure. This material is a block-type lambda trititanium pentoxide (block-type λ-Ti_3_O_5_). The block-type λ-phase accumulates a large heat energy of 237 kJ L^−1^ and exhibits a pressure-induced phase transition to beta trititanium pentoxide. The pressure-induced phase transition occurs by applying only several tens of bars, and half of the fraction transforms by 7 MPa (70 bar). Such a *low-pressure-responsive heat-storage ceramic* is effective to reuse excessive heat in automobiles or waste heat at industrial factories.

## Introduction

Automobiles, such as cars, trucks, and buses gain power using heat energy from burning fuel in an engine. Upon initiating the engine, an automobile consumes energy to warm the internal system to the appropriate temperature in order to start driving. On the other hand, excessive heat energy is generated and released into the atmosphere while driving. Fuel consumption would be reduced if this excessive heat energy could be used when restarting a car. Materials capable of accumulating heat energy, which are known as heat-storage materials, are classified into two categories: sensible heat-storage materials and solid–liquid latent heat-storage materials. The former includes bricks and concrete^1,2^, while the latter includes water, paraffin, and polyethylene glycol^3,4^. Regardless of the category, these materials release their accumulated heat energy over time. From this viewpoint, we focus on lambda trititanium pentoxide^5,6^, which is one of the external-stimuli-induced phase transition materials^7–23^. The accumulated energy in lambda trititanium oxide can be stored and released by an external pressure^24^. Such a heat-storage behavior cannot be observed in typical pressure-induced phase transitions^25–36^. Therefore, this material suggests potential applications in the industry^37,38^. More preferably, the pressure to extract the accumulated heat energy is desired to be less than 10 MPa (100 bar). Herein we develop an extremely low-pressure-responsive heat-storage ceramic composed of block-type λ-Ti_3_O_5_. This paper describes the material synthesis, crystal structure, and morphology of block-type λ-Ti_3_O_5_. Additionally, the low-pressure-induced phase transition, heat-storage process, thermal hysteresis loop, and the mechanism of the observed phenomenon in this material are reported.

## Results and Discussion

### Material, crystal structure, and morphology

**Figure 1:**
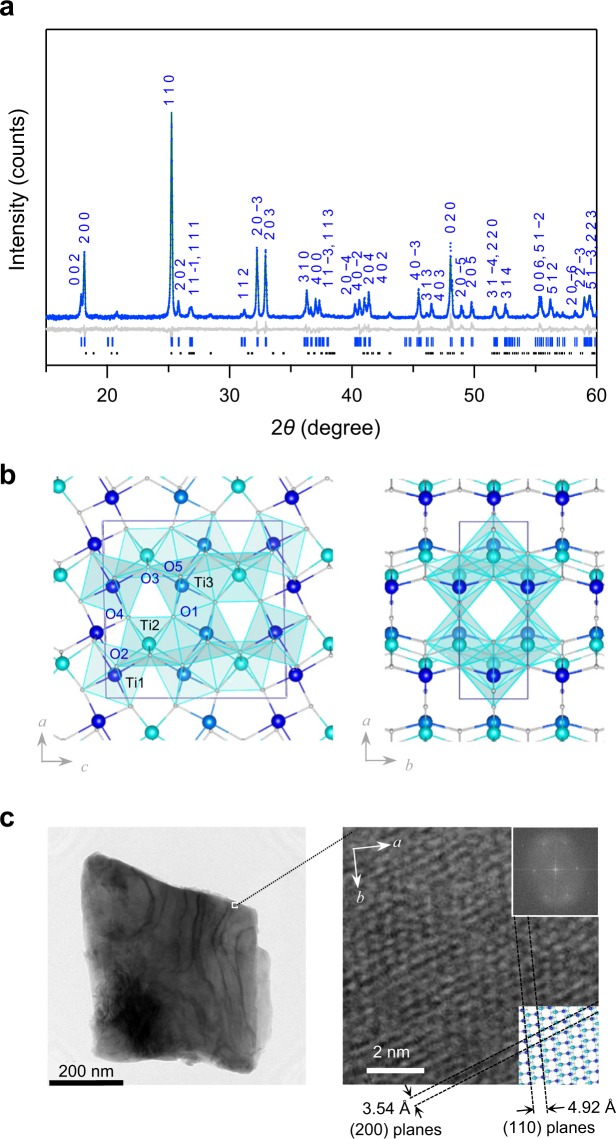
Morphology and crystal structure of block-type λ-Ti_3_O_5_. (**a**) XRPD pattern and Rietveld analysis of block-type λ-Ti_3_O_5_. (**b**) Crystal structure of block-type λ-Ti_3_O_5_ viewed along the *b*-axis (left) and *c*-axis (right). (**c)** TEM image of block-type λ-Ti_3_O_5_ (left) and enlarged figure of the TEM image with clear lattice fringes (right). Insets show the Fourier transform image (upper) and the atomic positions of the corresponding lattices (lower).

The target material was prepared by sintering the precursor rutile-type TiO_2_ at 1300 °C for 2 hours under a hydrogen atmosphere. The detail synthesis is described in the Methods section (Fig. S1). Elemental analysis by X-ray fluorescence (XRF) suggests that the formula of the obtained sample is Ti_3.00_O_5.00_ (Calc.: Ti 64.2%, O 35.8%; Found: Ti 64.1%, O 35.9%). The powder X-ray diffraction (PXRD) pattern with Rietveld analysis indicates a monoclinic crystal structure in the *C*2/*m* space group with lattice parameters of *a* = 9.8256(2) Å, *b* = 3.78889(4) Å, *c* = 9.9723(2) Å, and *β* = 91.2751(14)°. These features correspond to a crystal structure of λ-Ti_3_O_5_ (Fig. 1a,b, Table S1)^5^. In addition, the β-phase is included as a minor phase (monoclinic, *C*2/*m*, *a* = 9.7659(3) Å, *b* = 3.79907(7) Å, *c* = 9.4445(3) Å, *β* = 91.533(3)°). The morphology of the sample was investigated by transmission electron microscopy (TEM). The sample is comprised of block-shaped crystals of sub-micrometer length on a side (Figs 1c and S2). This crystal size is remarkably large compared to previously reported samples. According to the morphology of the primary particles, we call the present material *block-type lambda trititanium pentoxide* (block-type λ-Ti_3_O_5_).

### Release of heat energy by applying pressure to the λ-phase

**Figure 2:**
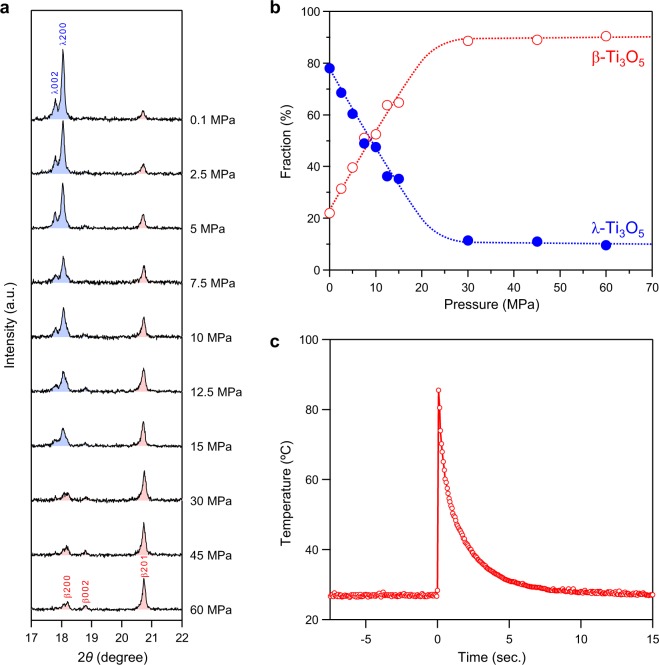
Pressure evolution of the phase fractions and pressure-induced heat release of block-type λ-Ti_3_O_5_. (**a**) Pressure evolution of the PXRD pattern. Blue and red indicate the peaks assigned to λ-Ti_3_O_5_ and β-Ti_3_O_5_, respectively. (**b**) Phase fractions of block-type λ-Ti_3_O_5_ (blue) and β-Ti_3_O_5_ (red) versus applied pressure. (**c**) Time dependence of the sample temperature on applying pressure as observed by thermography. Pressure is applied to the sample at *t* = 0.

The pressure effect on block-type λ-Ti_3_O_5_ was investigated by PXRD measurements after applying an external pressure (*P*) of 2.5, 5, 7.5, 10, 12.5, 15, 30, 45, 60, 230, or 600 MPa (Figs 2a and S3). As the pressure increases, the phase fraction of λ-Ti_3_O_5_ decreases while that of β-Ti_3_O_5_ increases (Fig. 2b). Above 30 MPa, the phase fractions show constant values. The transition pressure (*P*_1/2_), which is where the phase fractions of λ-Ti_3_O_5_ and β-Ti_3_O_5_ are equal, is 7 MPa.

### Thermograms before and after a pressure application

**Figure 3:**
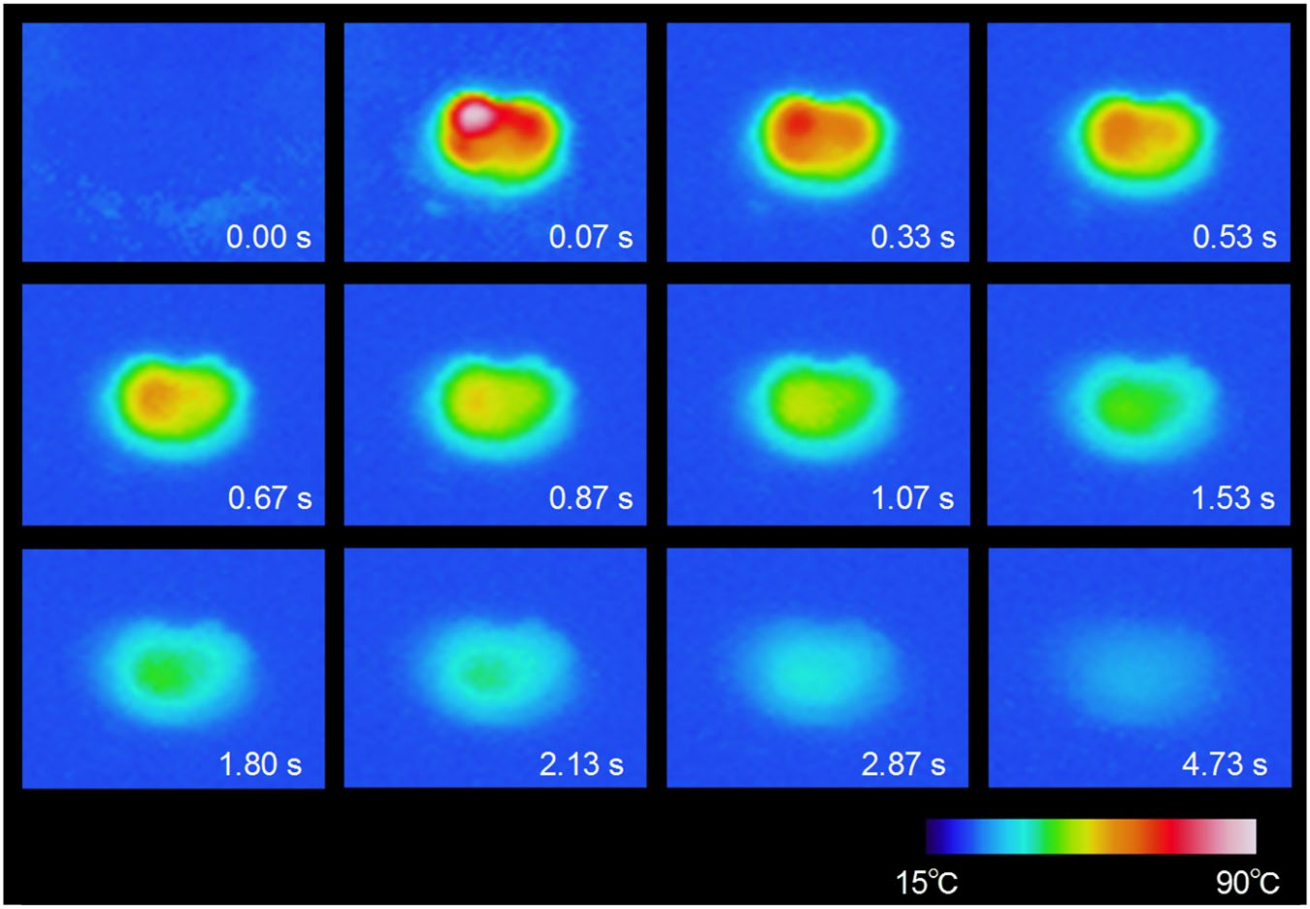
Time evolution of the thermographic image on applying pressure to block-type λ-Ti_3_O_5_. Snapshots taken from the thermogram of the sample on applying pressure as observed by thermography. Pressure is applied to the sample at *t* = 0. The sample temperature reached a maximum of 85.5 °C.

We visually measured the temperature change of the sample during a pressure-induced phase transition using thermography (Fig. 2c, Supplementary Movie S1). Pressure was applied by hitting the sample using a hammer. Initially, the temperature is 26.8 °C, and the thermal image is blue. Hitting the sample with a hammer instantly changes the thermal image color to white, which successively turns red, orange, yellow, green, and then back to blue (Fig. 3). The maximum temperature of the white area is 85.5 °C, indicating a temperature increase of 60 °C. The sample temperature reaches a maximum value in less than 67 ms after applying pressure, indicating that the heat energy is immediately released upon applying pressure. Then the temperature exponentially decreases with a decay time of 1.7 s.

We carried out the estimation of the pressure-released heat energy using thermography. Based on the heat capacity versus temperature curve of β-phase^24^, and by considering the temperature increase and the conversion ratio after hitting the sample with a hammer, the pressure-released energy was estimated to be 235 ± 7 kJ L^−1^. The details of the estimation process are described in Supplementary Section 6.

### Heat-storage process from the β- to λ-phase

**Figure 4:**
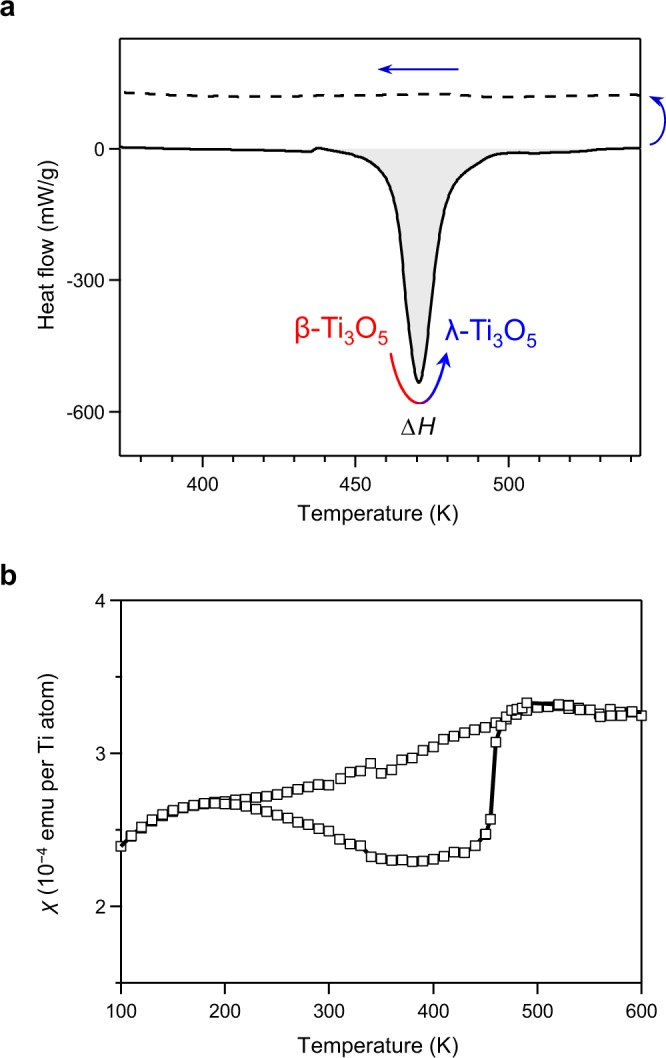
Heat-storage properties and thermal hysteresis loop of block-type λ-Ti_3_O_5_. (**a**) DSC chart of block-type λ-Ti_3_O_5_ with increasing temperature (solid line) and decreasing temperature (dotted line). (**b**) Temperature dependence of the magnetic susceptibility (*χ*) of block-type λ-Ti_3_O_5_.

The heat-storage temperature and accumulated heat energy were measured with a differential scanning calorimeter (DSC). For the measurement, the pressure-produced β-phase was used. In the initial heating process from room temperature to 600 K, an endothermic peak (i.e., heat-storage peak) is observed at 471 K (198 °C). Analyses of the DSC curve shows that the accumulated heat energy is 237 kJ L^−1^ (Fig. 4a). Conversely, in the cooling process from 600 K to 300 K, an exothermic peak (i.e., heat-release peak) is not observed. These data indicate that λ-Ti_3_O_5_ stores the latent heat energy of 237 kJ L^−1^.

### Thermal hysteresis loop of the phase transition between the β- and λ-phases

To investigate the origin of such a low pressure–induced phase transition, we measured the temperature dependence of the magnetic susceptibility (*χ*) of block-type λ-Ti_3_O_5_ using a superconducting quantum interference device (SQUID) magnetometer (Figs 4b and S4). In the cooling process from 600 K, the *χ* value remains nearly constant around 0.0003 emu per Ti atom, gradually decreases below 150 K, but rapidly increases below 30 K. In the heating process from 2 K to 190 K, the *χ* values are the same as those in the cooling process. In the heating process around 190 K, the *χ* value begins to diverge; it takes lower values but abruptly increases at 455 K until it returns to the original values. A thermal hysteresis loop is observed with a branch point in the low temperature region (*T*_L_) of 190 K and a closing point in the high temperature region (*T*_H_) of 455 K. The temperature width of the thermal hysteresis (*ΔT* ≡ *T*_H_−*T*_L_) is 265 K. Such a thermal hysteresis loop has not been observed in the previous λ-Ti_3_O_5_. It should be noted that the *χ* value of ~0.0003 emu per Ti atom in the cooling process indicates Pauli paramagnetism. The gradual decrease below 150 K is due to the spin–orbital interaction of the Ti^3+^ ions, while the increase below 30 K is attributed to the Curie paramagnetic component due to lattice defects.

### Mechanism of the appearance of thermal hysteresis loop and low pressure–induced heat energy release

**Figure 5:**
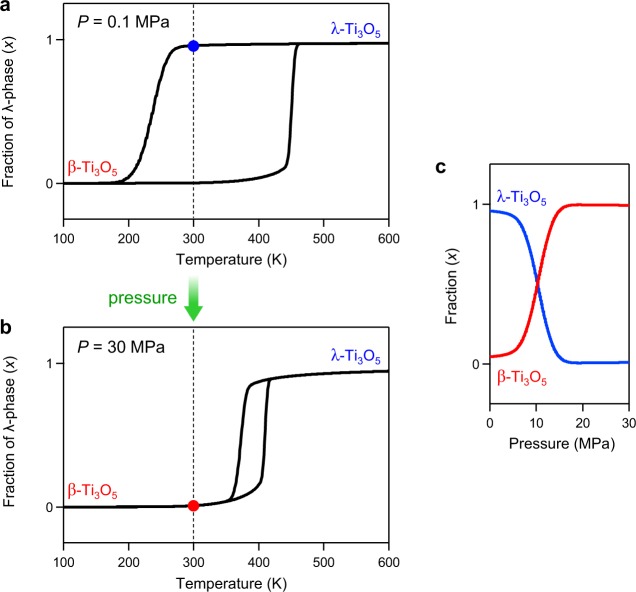
Mechanism of the pressure-induced phase transition based on the Slichter-Drickamer mean-field model. (**a**,**b**) Calculated λ-phase fraction (*x*) versus temperature curves at *P* = 0.1 MPa (**a**) and 30 MPa (**b**). Calculations were performed for the cooling process and the heating process under the conditions of Δ*H* = 13.7 kJ mol^−1^, Δ*S* = 34.6 J K^−1^ mol^−1^, *γ*_b_ = −2.4 J K^−1^ mol^−1^, and *γ*_c_ = −0.12 kJ MPa^−1^ mol^−1^, while assuming the γ_a_ value has a normal distribution centered at 12.88 kJ mol^−1^ with a standard deviation of 0.3 kJ mol^−1^. (**c**) Calculated λ-phase and β-phase fractions versus pressure at 300 K.

Next we considered the origin of the thermal hysteresis between the λ- and β-phases and the phase transition with an extremely weak pressure using thermodynamic analysis based on the Slichter and Drickamer mean-field model (SD model) (see Methods)^39^. In the SD model, the Gibbs free energy (*G*) of the system is described as *G* = *x*Δ*H* + *γ x*(1 − *x*) + *T*{*R*[*x* ln *x* + (1 − *x*)ln(1 − *x*)] − *x*Δ*S*} + *G*_β_ with the Gibbs free energy of β-phase (*G*_β_) as the standard, and the interaction parameter (*γ*) between the λ- and β-phases related to the elastic interactions inside the crystal is defined by *γ* = *γ*_a_ + *γ*_b_(*T*) + *γ*_c_(*P*). From the result of the DSC measurement, the transition enthalpy (Δ*H*) is 13.7 kJ mol^−1^. When the transition entropy (Δ*S*) and interaction parameters are set as a particular combination of values, the SD model calculation well reproduces the observed thermal hysteresis; i.e., the phase transitions of β-phase → λ-phase and λ-phase → β-phase occur at *T*_L_ of 194 K and *T*_H_ of 458 K, respectively, at a pressure of 0.1 MPa (1 bar) (Fig. S5a, black line). Hence, the thermal hysteresis loop appears due to the existence of the energy barrier between two bistable phases with close energy states. Furthermore, by assuming the *γ* value has a distribution due to the inhomogeneity of the primary crystal size, the SD model calculation well reproduces the observed hysteresis loop as shown in Fig. 5a, where the transition in the cooling process is gradual and that in the heating process is abrupt.

Next, we calculated the pressure dependence of the λ-Ti_3_O_5_ phase fraction. Applying pressure to the system causes the energy barrier to disappear and induces a phase transition from λ-Ti_3_O_5_ to β-Ti_3_O_5_ (Fig. 5b). Figure S5b shows the *x* vs. *T* plots at *P* = 30 MPa. The pressure dependence of *x* shows a threshold in the pressure-induced phase transition. By considering the distribution of the *γ* value (Fig. S5c), a gradual pressure-induced phase transition as in Fig. 2b is well reproduced (Fig. 5c).

## Conclusion

**Figure 6:**
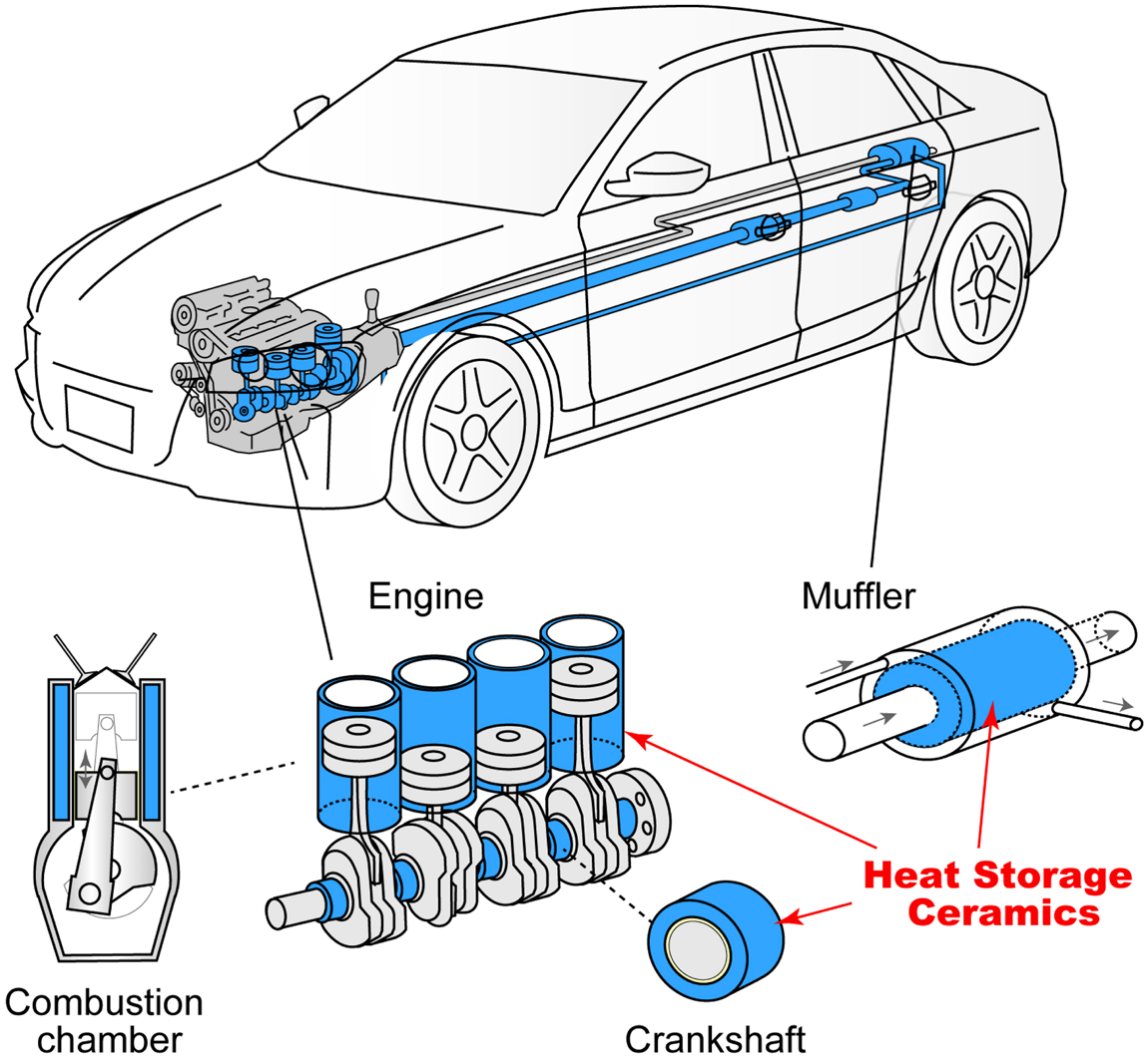
Possible applications of block-type λ-Ti_3_O_5_ for automobiles. Schematic image of where block-type λ-Ti_3_O_5_ could be applied as a heat-storage material in an automobile. Blue areas indicate the possible components to use the heat-storage material: combustion chamber, crankshaft, and muffler.

Herein we report a newly developed heat-storage ceramic based on block-type λ-Ti_3_O_5_, which preserves the heat energy for a long period and shows a low pressure–induced heat energy release. Block-type λ-Ti_3_O_5_ accumulates a large latent heat energy of 237 kJ L^−1^, and the accumulated heat energy can be extracted by applying an extremely weak pressure of only several MPa with a *P*_1/2_ value of 7 MPa. For example, the pressure of a compressed gas cylinder is in the range from 12 to 30 MPa^40^, suggesting that the present material could be triggered using a gas cylinder. The long-term heat-storage property of block-type λ-Ti_3_O_5_ and its release of accumulated heat energy by low pressure originate from the bistability (λ-phase and β-phase) of the present material and the existence of an energy barrier between the two phases. Due to the energy barrier, block-type λ-Ti_3_O_5_ exhibits one of the largest thermal hysteresis loops among condensed matter with a Δ*T* value of 265 K. Because the energy barrier disappears under weak pressure, the λ-phase transforms into the β-phase and releases the accumulated latent heat energy, which is comparable to the latent heat energies of solid–liquid phase-transition materials, e.g., water (320 kJ L^−1^), paraffin (140 kJ L^−1^), and polyethylene glycol (165 kJ L^−1^). The energy barrier is attributed to the elastic interaction within the material. The behaviors of the temperature dependence and pressure dependence of block-type λ-Ti_3_O_5_ are well reproduced by thermodynamic calculations. From the viewpoint of automobile applications, transition pressures below 10 MPa are preferable. Therefore, the present heat-storage ceramic should be useful in automobile components near engines and mufflers (Fig. 6)^37,38^, since the heat-storage ceramic can warm the cooled internal system when restarting the automobile. Additionally, an example of other possible applications is solar power plants. In solar power plants, nitrates have been used in the heat storage tanks. Since the present material has both properties of long-term latent heat storage and sensible heat storage, it is expected to be useful for the heat storage system at solar power plants (Fig. S6).

## Methods

### Physical measurements

Elemental analysis of the prepared sample was performed using X-ray fluorescence spectroscopy (Rigaku, ZSX PrimusII). TEM measurements were conducted using a JEOL JEM-2000EXII and JEM-4000FXII. The XRD measurements were conducted by a Rigaku Ultima IV with Cu Kα radiation (*λ* = 1.5418 Å). Rietveld analyses were performed by the RIETAN-FP program. The magnetic properties were measured using a superconducting quantum interference device (SQUID) magnetometer (Quantum Design, MPMS 7). DSC was performed on a Rigaku DSC 8230.

### Thermodynamic analysis

In the Slichter and Drickamer mean-field model, the Gibbs free energy of the system is described as *G* = *x*Δ*H* + *γ x*(1 − *x*) + *T*{*R*[*x* ln *x* + (1 − *x*)ln(1 − *x*)] − *x*Δ*S*} + *G*_β_, where *x* is the ratio of the λ-phase. Δ*H* and Δ*S* are the transition enthalpy and transition entropy for the transition between the λ- and the β-phases, respectively. *γ* is the interaction parameter between the λ- and β-phases. *G*_β_ is the Gibbs free energy of the β-phase, which is set as the origin of the energies, and *R* is the gas constant. The *γ* value depends on the temperature and pressure; *i.e*., *γ* = *γ*_a_ + *γ*_b_(*T*) + *γ*_c_(*P*). The observed phase transition is due to the metal–semiconductor phase transition between charge-delocalized λ-Ti_3_O_5_ and charge-localized β-Ti_3_O_5_, which are regarded as Ti(1)^3.3+^–Ti(2)^3.3+^–Ti(3)^3.3+^ and Ti(1)^3.0+^–Ti(2)^3.7+^–Ti(3)^3.3+^, respectively. From the DSC measurement result, the Δ*H* value is 13.7 kJ mol^−1^. When the Δ*S* value and the interaction parameters are set as follows: Δ*S* = 34.6 J K^−1^ mol^−1^, *γ*_a_ = 13.48 kJ mol^−1^, *γ*_b_ = −2.4 J K^−1^ mol^−1^, and *γ*_c_ = −0.12 kJ MPa^−1^ mol^−1^, the SD model calculations indicated thermal hysteresis loops as shown in Fig. S5a,b (black lines). Furthermore, by assuming that the *γ*_a_ value has a normal distribution centered at 12.88 kJ mol^−1^ with a standard deviation of 0.3 kJ mol^−1^ (Fig. S5c), the SD model calculation qualitatively reproduces the observed thermal hysteresis loop. The pressure dependence of *x* was also calculated using the same parameters.

## Supplementary information

Supplementary Information

Supplementary Movie S1

## Data Availability

All data are available upon reasonable request from the corresponding authors.
