# Supplementary material for: Low-pressure-responsive heat-storage ceramics for automobiles
Source: Sci Rep. 2019 Sep 18;9:13203. doi: 10.1038/s41598-019-49690-0 (PMC6751172; doi:10.1038/s41598-019-49690-0)
Supplement: Supplementary file 1 — Supplementary Information [file 41598_2019_49690_MOESM1_ESM.pdf]

Low-pressure-responsive heat-storage ceramics for automobiles

Shin-ichi Ohkoshi[1,*], Hiroko Tokoro[1,2], Kosuke Nakagawa[1], Marie Yoshikiyo[1], Fangda Jia[1], and Asuka Namai[1]

[1] *Department of Chemistry, School of Science, The University of Tokyo, 7-3-1 Hongo, Bunkyo-ku, Tokyo 113-0033, Japan*
[2] *Division of Materials Science, Faculty of Pure and Applied Sciences, University of Tsukuba 1-1-1 Tennodai, Tsukuba, Ibaraki 305-8577, Japan*

*Correspondence should be addressed to S. O.
ohkoshi@chem.s.u-tokyo.ac.jp

Table of Contents: **Page**

Section 1.	Legend for Supplementary Movie		S2
Section 2.	Synthesis	Fig. S1	S3
Section 3.	Crystal structure analysis of block-type λ-Ti_3O_5	Table S1	S4
Section 4.	TEM images of block-type λ-Ti_3O_5	Fig. S2	S5
Section 5.	Pressure induced phase transition in block-type λ-Ti_3O_5	Fig. S3	S6
Section 6.	Released heat energy by pressure-induced phase transition		S9
Section 7.	Magnetic properties of block-type λ-Ti_3O_5	Fig. S4	S10
Section 8.	Thermodynamic analysis based on the SD model	Fig. S5	S11
Section 9.	Possibility of heat-storage ceramics for solar power plants	Fig. S6	S12

Section 1. Legend for Supplementary Movie

Supplementary Movie S1 | Pressure-induced heat release of block-type λ-Ti_3O_5 observed by thermography. Initial part of the movie shows the experimental setup. Upper left photograph shows the overall setup of hitting the sample in front of a thermography camera. Lower right photograph shows an enlarged view of the sample. Second part of the movie is the time evolution of the thermogram of the enlarged sample area. The time is indicated in the lower right, and the pressure was applied at t = 0 s. The following part shows the snapshots of the thermogram. The final part of the movie shows the time dependence of the sample temperature, indicating an increase of temperature by 60 ºC induced by pressure application.

Section 2. Synthesis

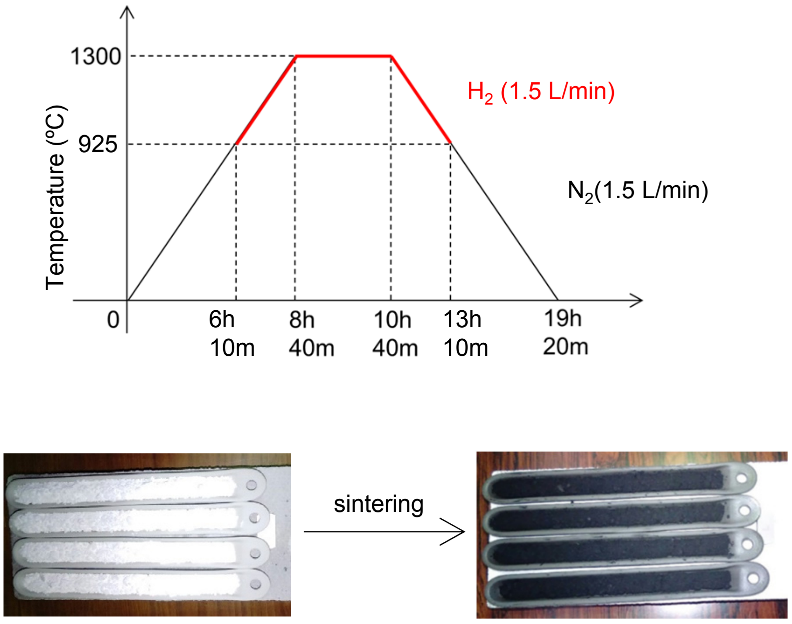

Supplementary Fig. S1 | Synthesis of block-type λ-Ti_3O_5. Sintering process and photos of the sample before and after sintering.

Section 3. Crystal structure analysis of block-type λ-Ti_3O_5

Supplementary Table S1 | Structural parameters obtained from Rietveld analysis of the XRPD pattern of block-type λ-Ti_3O_5.

Polymorph		λ-Ti_3O_5	
Crystal system		Monoclinic	
Space group		*C*2/*m* (No. 12)	
a (Å)		9.8256(2)	
b (Å)		3.78889(4)	
c (Å)		9.9723(2)	
β (°)		91.2751(14)	
V (Å^3)		371.155(11)	
Z		4	
R_{wp} (%)		5.85	
S		1.48	
	x/*a*	*y*/*b*	*z*/*c*
Ti(1)	0.6310(3)	0	0.0511(2)
Ti(2)	0.3051(2)	0	0.2448(3)
Ti(3)	0.6357(3)	0	0.4353(2)
O(1)	0.4582(9)	0	0.3834(7)
O(2)	0.1785(8)	0	0.0618(8)
O(3)	0.7405(8)	0	0.2499(8)
O(4)	0.5462(8)	0	0.8682(7)
O(5)	0.1850(8)	0	0.4279(8)

Section 4. TEM images of block-type λ-Ti_3O_5

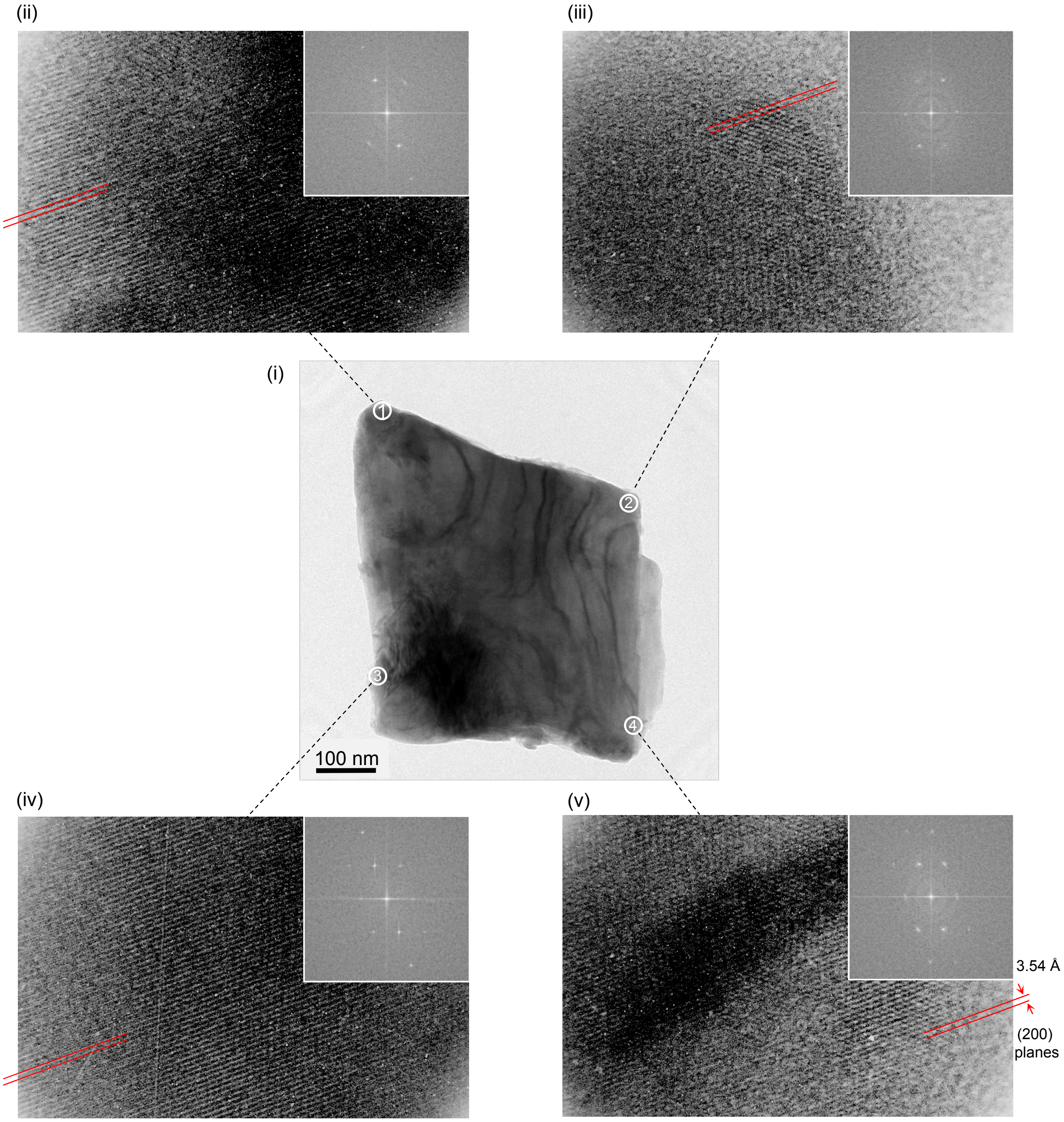

Supplementary Fig. S2 | (i) TEM image of block-type λ-Ti_3O_5 and (ii)–(v) enlarged images of the TEM image with clear lattice fringes. Insets show the Fourier transform images.

Section 5. Pressure induced phase transition in block-type λ-Ti_3O_5

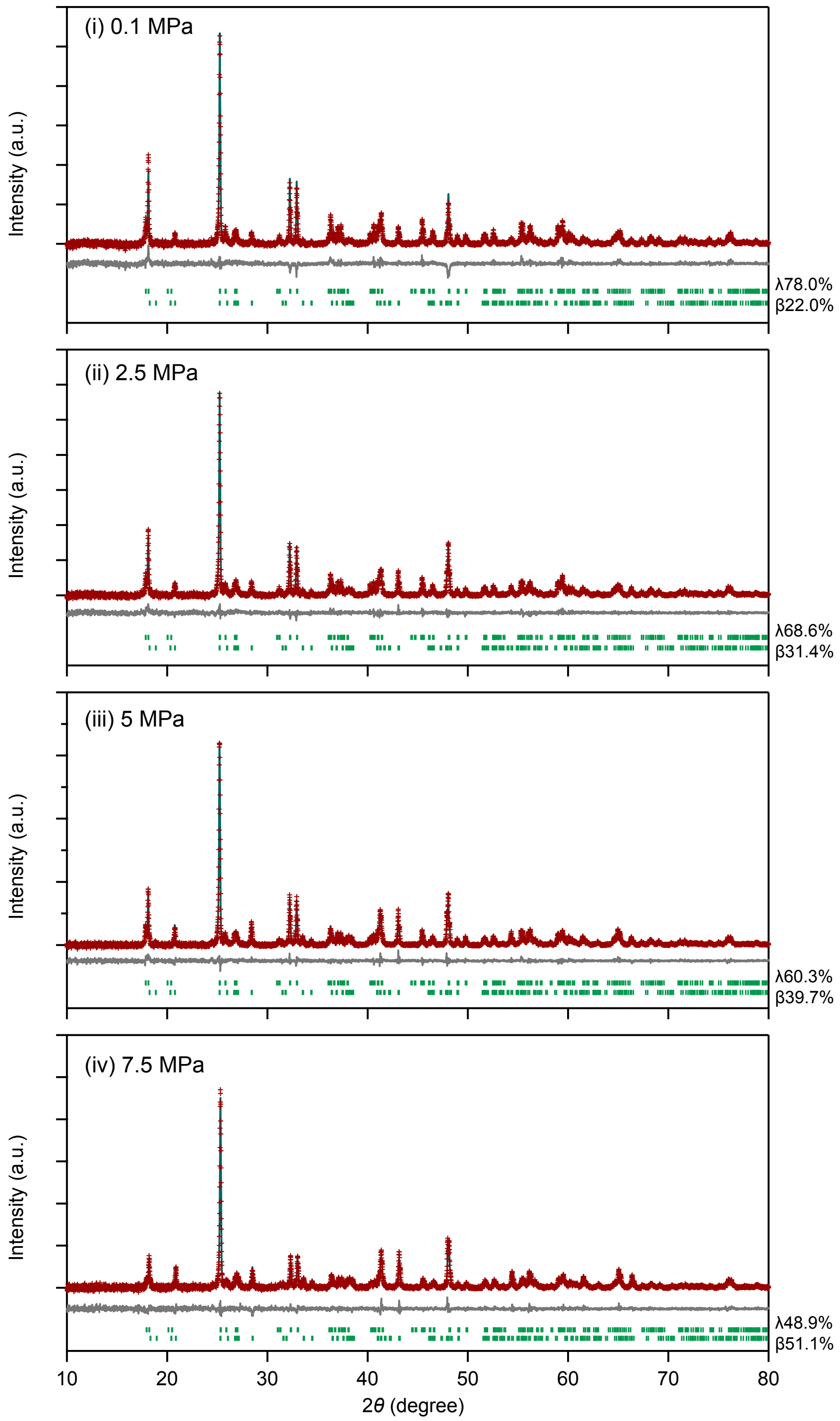

Supplementary Fig. S3-1 | Rietveld analyses of the XRPD patterns. PXRD patterns with Rietveld analysis under (i) atmospheric pressure (0.1 MPa), and after applying the pressures of (ii) 2.5 MPa, (iii) 5 MPa, and (iv) 7.5 MPa. Red plots, blue line, and gray line are the observed pattern, total calculated pattern, and residual pattern, respectively. Green bars represent the calculated positions of the Bragg reflections of the λ-phase and β-phase.

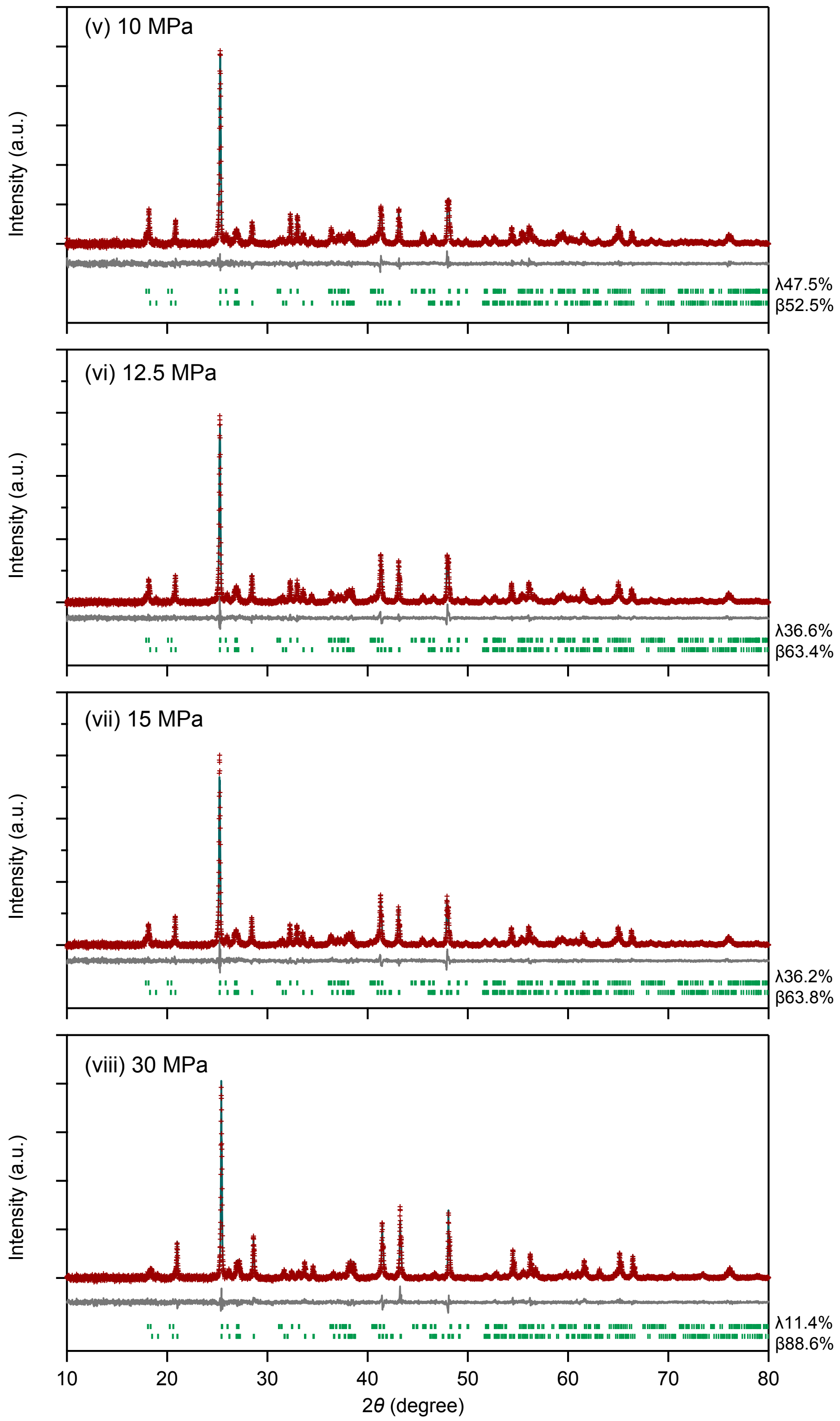

Supplementary Fig. S3-2 | Rietveld analyses of the XRPD patterns. PXRD patterns with Rietveld analysis of the sample after applying the pressures of (v) 10 MPa, (vi) 12.5 MPa, (vii) 15 MPa, and (viii) 30 MPa. Red plots, blue line, and gray line are the observed pattern, total calculated pattern, and residual pattern, respectively. Green bars represent the calculated positions of the Bragg reflections of the λ-phase and β-phase.

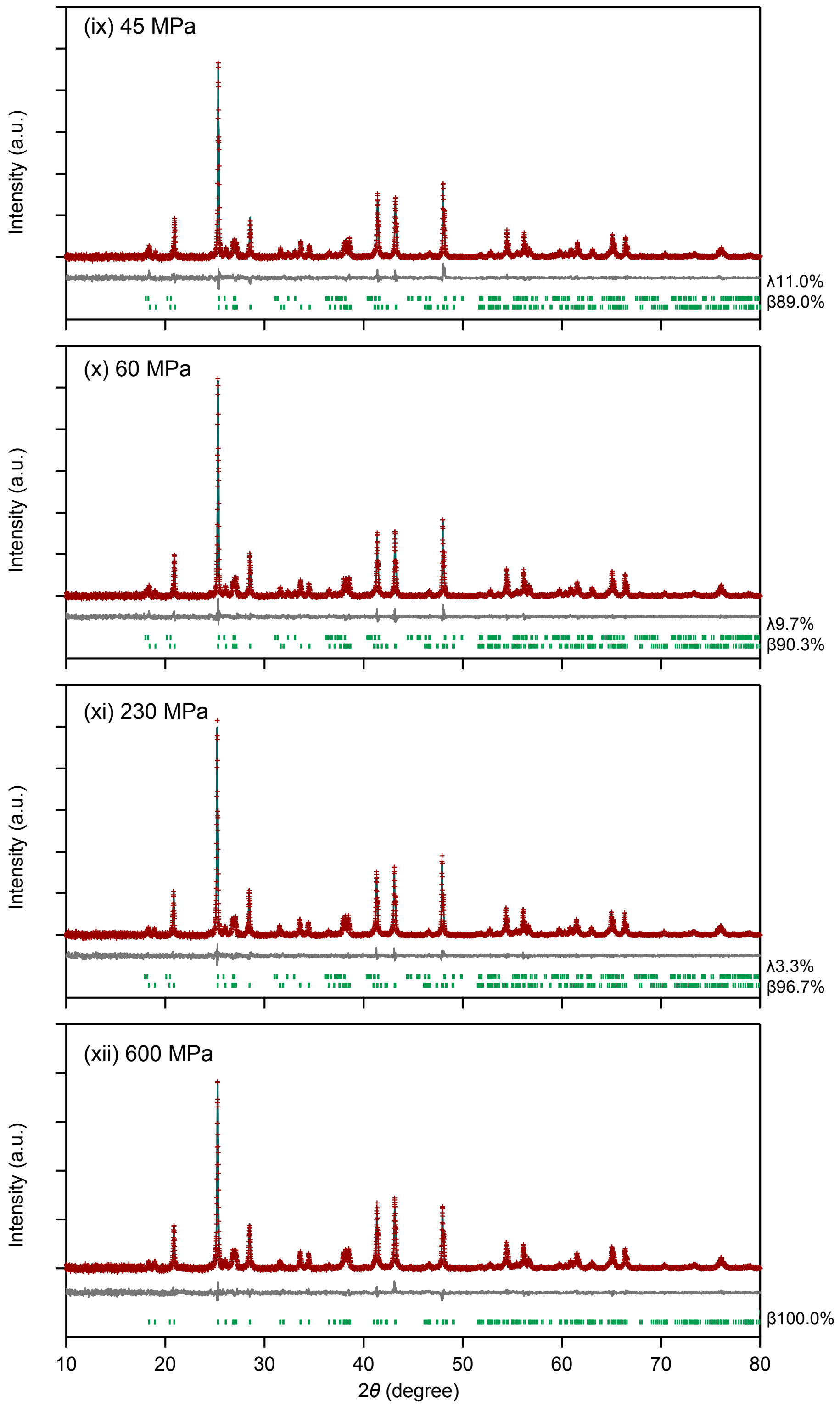

Supplementary Fig. S3-3 | Rietveld analyses of the XRPD patterns. PXRD patterns with Rietveld analysis of the sample after applying the pressures of (ix) 45 MPa, (x) 60 MPa, (xi) 230 MPa, and (xii) 600 MPa. Red plots, blue line, and gray line are the observed pattern, total calculated pattern, and residual pattern, respectively. Green bars represent the calculated positions of the Bragg reflections of the λ-phase and β-phase.

Section 6. Released heat energy by pressure-induced phase transition

To estimate the pressure-released heat energy, we measured the increase of the temperature by pressure application using thermography. The rate of heat release after hitting the sample with a hammer (pressure application) is much faster than the heat exchange rate from the sample to the surroundings. Therefore, we consider the system as adiabatic during the pressure-induced phase transition. In the adiabatic system, the released heat energy corresponds to the increase of enthalpy ($\Delta H'$) of the β-phase, i.e., $\Delta H' = \int C_P(T)\mathrm{d}T$, where $C_P(T)$ is the temperature dependence of the heat capacity. In our previous paper,[24] $C_P(T)$ of β-Ti_3O_5 was well reproduced by the following two-Debye model:

$$C_P(T) = \sum_{i=1}^{2} 9Rc_i(T/\theta_i)^3 \int_0^{\theta_i/T} x^4 e^x/(e^x-1)^2 dx \ ,$$

where R is the gas constant, c_i is coefficient, θ_i is the Debye temperature, x is $\hbar\omega/k_B T$, $\hbar$ is the reduced Planck constant, ω is phonon frequency, and k_B is Boltzmann constant, with constant parameters of c_1 = 2.7(1), c_2 = 5.8(1), θ_1 = 4.3(1) × 10^2 K, and θ_2 = 9.3(2) × 10^2 K. Considering the conversion ratio from λ-phase to β-phase and the temperature increase after hitting the sample with a hammer, the pressure-released heat energy of this system was estimated to be 235 ± 7 kJ L^{-1}.

Section 7. Magnetic properties of block-type λ-Ti_3O_5

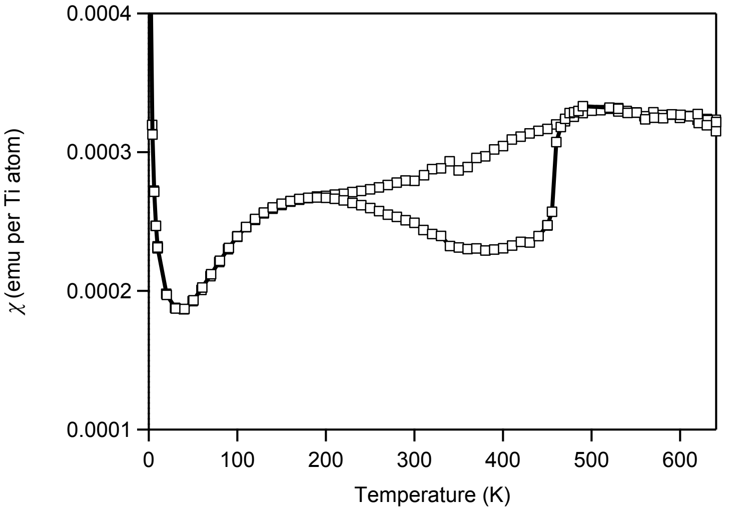

Supplementary Fig. S4 | Temperature dependence of the magnetic susceptibility (χ) of block-type λ-Ti_3O_5. χ versus T graph of the block-type λ-Ti_3O_5 measured in the cooling and heating process for the temperature region of 10–640 K under an external field of 0.5 T.

Section 8. Thermodynamic analysis based on the SD model

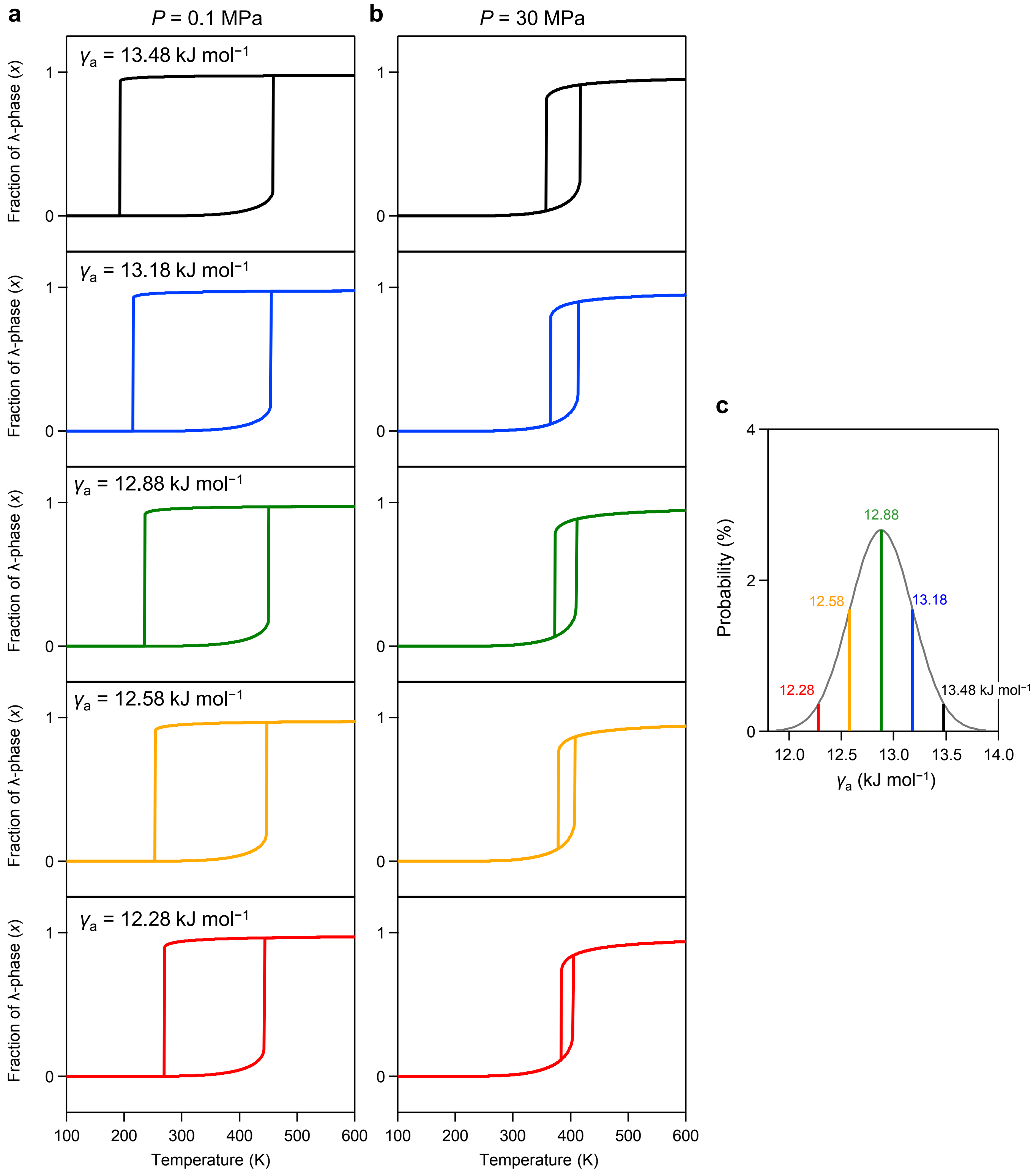

Supplementary Fig. S5 | a,b, Calculated λ-phase fraction (x) versus temperature curves at (**a**) P = 0.1 MPa and (**b**) 30 MPa at various γ_a value of 13.48 kJ mol^{-1} (black), 13.18 kJ mol^{-1} (blue), 12.88 kJ mol^{-1} (green), 12.58 kJ mol^{-1} (orange), and 12.28 kJ mol^{-1} (red). **c**, The distribution of the γ_a value. Other parameters were set as ΔH = 13.7 kJ mol^{-1}, ΔS = 34.6 J K^{-1} mol^{-1}, γ_b = −2.4 J K^{-1} mol^{-1}, and γ_c = −0.12 kJ MPa^{-1} mol^{-1}.

Section 9. Possibility of heat-storage ceramics for solar power plants

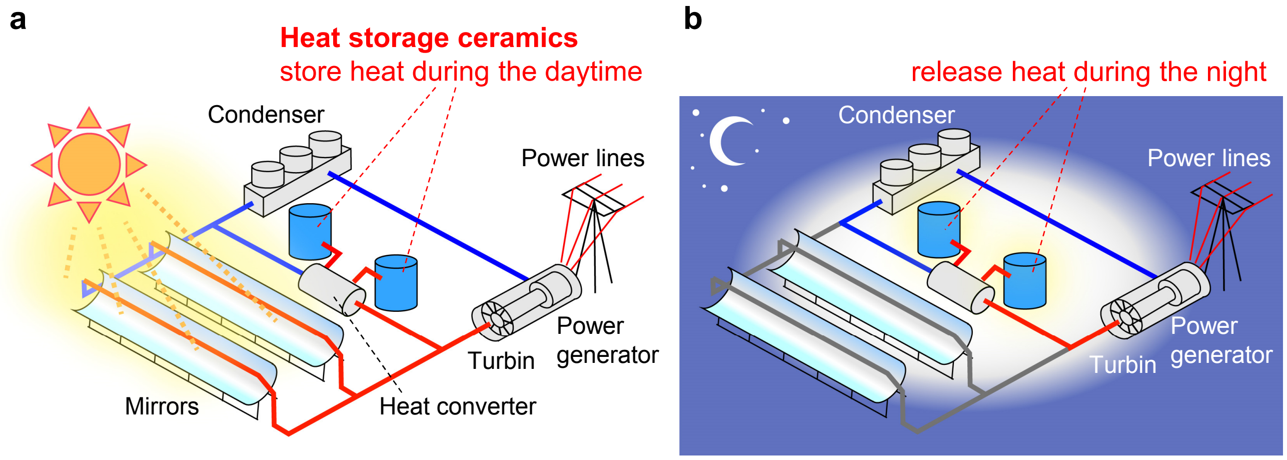

Supplementary Fig. S6 | Possibility of heat-storage ceramics for solar power plants. **a**, During the daytime, solar heat is used to generate electricity and to store heat in the heat storage ceramic placed in the heat storage tanks (β-phase → λ-phase). **b**, At night, pressure is applied to the heat storage ceramic in the heat storage tanks to release thermal energy (λ-phase → β-phase), which is used to generate electricity.
